# Cutaneous Vasculopathy in a COVID-19 Critically Ill Patient: A Histologic, Immunohistochemical, and Electron Microscopy Study

**DOI:** 10.1155/2021/6644853

**Published:** 2021-04-03

**Authors:** Anna Kyriakoudi, Konstantinos Pontikis, Athanasios Tsaraklis, Efthymia Soura, Christine Vourlakou, Athanasios Kossyvakis, Efstathia Potamianou, Evangelos Kaniaris, Iliana Ioannidou, Andreas Mentis, Ismini Kloukina, Maria Daganou, Antonia Koutsoukou

**Affiliations:** ^1^ICU, 1st Department of Respiratory Medicine, National and Kapodistrian University of Athens Medical School, “Sotiria” Hospital, Athens, Greece; ^2^Dermatology Department, “Sotiria” Hospital, Athens, Greece; ^3^1st Department of Dermatology-Venereology, National and Kapodistrian University of Athens, Andreas Sygros Hospital, Athens, Greece; ^4^Pathology Department, Evangelismos General Hospital, Athens, Greece; ^5^Public Health Laboratories, Hellenic Pasteur Institute, Athens, Greece; ^6^Center of Basic Research, Biomedical Research Foundation Academy of Athens, Athens, Greece

## Abstract

We describe a critically ill, SARS-CoV-2 positive patient with respiratory failure and thrombotic/livedoid skin lesions, appearing during the course of the disease. The biopsy of the lesions revealed an occlusive, pauci-inflammatory vasculopathy of the cutaneous small vessels characterized by complement and fibrinogen deposition on vascular walls, pointing to a thrombotic vasculopathy. Transmission electron microscopy of the affected skin failed to reveal any viral inclusions. Clinical evaluation and laboratory findings ruled out systemic coagulopathies and disseminated intravascular coagulation, drug-induced skin reaction, and common viral rashes. Our hypothesis is that the, herein evidenced, microvascular occlusive injury might constitute a significant pathologic mechanism in COVID-19, being a common denominator between cutaneous and pulmonary manifestations.

## 1. Introduction

At the end of 2019, a cluster of interstitial pneumonia cases were reported in Wuhan, China, later discovered to be caused by a novel coronavirus, subsequently termed severe acute respiratory syndrome coronavirus-2 (SARS-CoV-2). Cases rapidly increased worldwide, and on March 12, 2020, the World Health Organization declared a pandemic [1].

The most common clinical manifestation of SARS-CoV-2 infection, termed coronavirus disease-2019 (COVID-19), is pneumonia. In addition to lower respiratory system insult, gastrointestinal symptoms have also been reported, while headache, sore throat, rhinorrhea, and anosmia are less frequent [2]. Currently, there are few and conflicting data in the scientific literature regarding COVID-19 associated with skin manifestations. In a recent review, describing the clinical characteristics of the disease in China, a rash was observed in 0.2% of cases [3]. This percentage seems to vary greatly; Recalcati et al. reported that up to 20.4% of patients develop cutaneous lesions [4], while others place that percentage around 4%, or even less [5]. Numerous case reports have reported petechiae, retiform purpura, livedo racemosa, morbilliform or vesicular eruptions, and urticaria-type or chilblain-type lesions [4, 6, 7]. A similar cluster of morphologies, namely, purpuric/vasculitic lesions, was proposed by Marzano et al., thus, simplifying the distinction into two basic morphologic entities, inflammatory/exanthematous rashes, and vasculopathic/vasculitic eruptions [8]. At this point, the pathogenesis and clinical importance of COVID-19 cutaneous symptoms is still uncertain [7]. Our case illustrates the vasculopathic cutaneous consequences of SARS-CoV-2 infection and proposes a possible prothrombotic connection between lung and skin disease [9].

## 2. Case Report

A 79-year-old man was admitted in the hospital on March 19, 2020, due to fever and dyspnea, lasting 3 days. His medical history was significant for hypertension, type 2 diabetes, hyperuricemia, and benign prostatic hyperplasia. He reported close contact with a confirmed COVID-19 case. SARS-CoV-2 RNA was detected in an upper respiratory tract specimen by reverse transcriptase-polymerase chain reaction (RT-PCR) testing. He presented mild respiratory failure, and oxygen therapy was instituted via a nasal canula at 2-3 L/min. Chest CT scan revealed peripherally distributed, bilateral ground glass, and consolidative opacities. He was prescribed hydroxychloroquine, azithromycin, and empiric ceftaroline. Despite treatment, respiratory failure progressed, and four days later, he was transferred to the intensive care unit (ICU). Upon ICU admission, he failed a short course of high flow nasal canula oxygen therapy (FiO2 80%-100% at a flow rate of 60 L/min), and he underwent orotracheal intubation. Multiplex PCR–testing (BIOFIRE® FILMARRAY® Pneumonia Plus Panel) performed on bronchial secretions did not detect common bacterial or viral respiratory pathogens.

Following mechanical ventilation initiation, he developed shock and received high doses of norepinephrine and argipressin, along with hydrocortisone, at a dose of 200 mg daily. At this point, heparin was prescribed due to concurrent paroxysmal atrial fibrillation.

**Figure 1:**
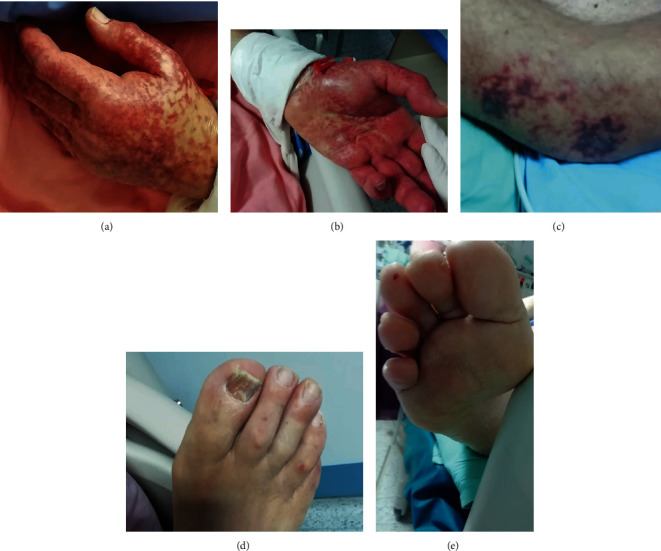
Initial lesions of retiform purpura or livedo on dorsal (a) and palmar (b) area of hands, and elbows (c). Scattered petechial lesions on toes on dorsal (d) and plantar (e) area of toes.

Upon shock recovery and withdrawal of vasopressors and hydrocortisone, on March 26, 2020 (four days since ICU admission and 10 days since symptom onset), cutaneous lesions were noted. These lesions included retiform purpura located in the patient's hands, livedo racemosa in elbows, and a mild purpuric rash, bilaterally on the trunk and plantar areas (Figure 1). Information as to whether the lesions were pruritic could not be obtained, due to sedation. No local pressure had been applied on the affected areas. Laboratory findings were of no clinical significance (moderately elevated leukocyte and platelet count, normal prothrombin and activated partial thromboplastin times, and normal plasma fibrinogen concentration and moderately elevated D-Dimers). A complete autoimmune marker panel was negative (ANA, anti-ds-DNA, c-ANCA, p-ANCA, anti-MPO, anti-PR3, and rheumatoid factor). A punch biopsy was performed, and two skin tissue samples were obtained, 5 mm in diameter, from the dorsal area of both hands.

**Figure 2:**
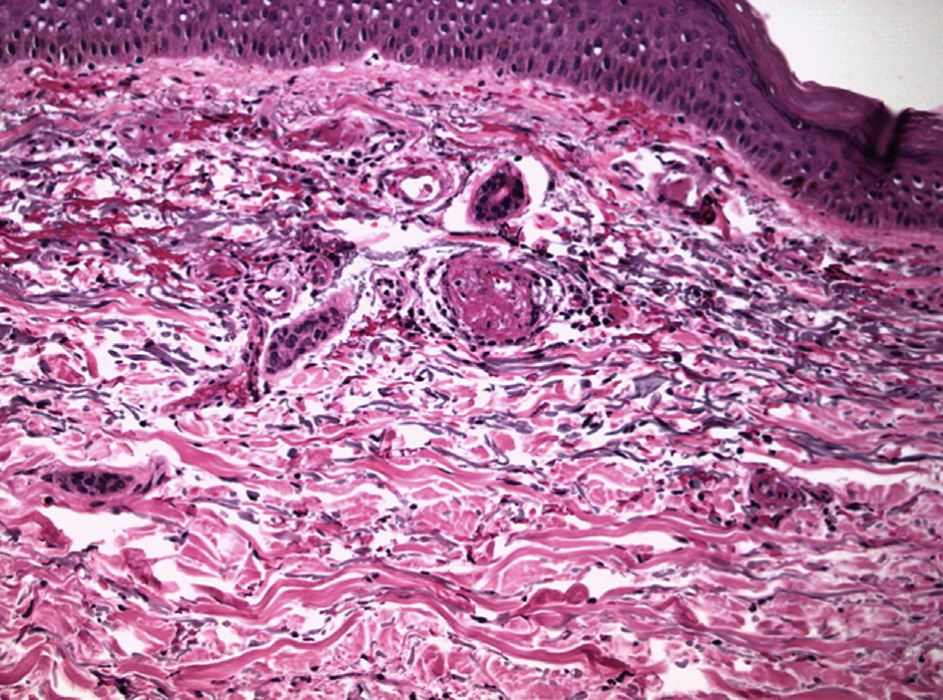
Endovascular fibrin clots. Generalized inflammation with a mainly perivascular pattern.

**Figure 3:**
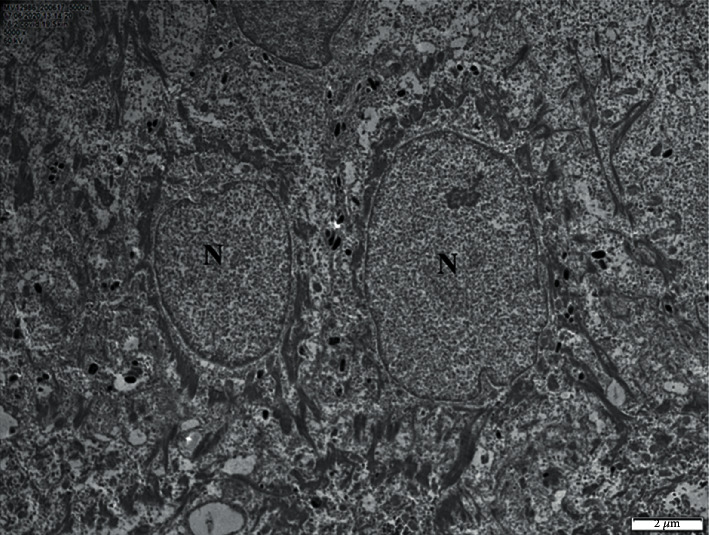
Representative transmission electron micrographs from skin biopsy illustrating the ultrastructure of typical keratinocytes in the stratum spinosum. *Ν* represent nucleus. No structures consistent with viral inclusions were found.

Histologic examination revealed the presence of occlusive vasculopathy of cutaneous microvessels, resembling disseminated intravascular coagulation, with minimal inflammation and no apparent vasculitis (Figure 2). Direct immunofluorescence showed the deposition of IgA, IgM, C1q, C3, C4, and fibrinogen on the endovascular fibrin clots. Transmission electron microscopy (TEM) was additionally performed on the same, formalin-fixed, paraffin-embedded biopsy block. Tissue cubes of 1 mm^3^ were cut with a razor blade and reprocessed, according to a protocol described by Graham and Orenstein [10]. After deparaffinization of skin biopsy blocks, small pieces of tissue were processed for conventional transmission electron microscopy. Specimens were fixed overnight in 2.5% glutaraldehyde, postfixed in osmium tetroxide, and embedded in epoxy resins. Thin sections were stained with uranyl acetate and lead citrate and subsequently observed with a Philips TEM 420 Electron Microscope. No viral inclusion structures were detected in the examined skin areas, including keratinocytes and endothelial cells (Figure 3). A SARS-CoV-2 specific RT-PCR was performed on skin samples, and the virus nucleic acid was not detected.

**Figure 4:**
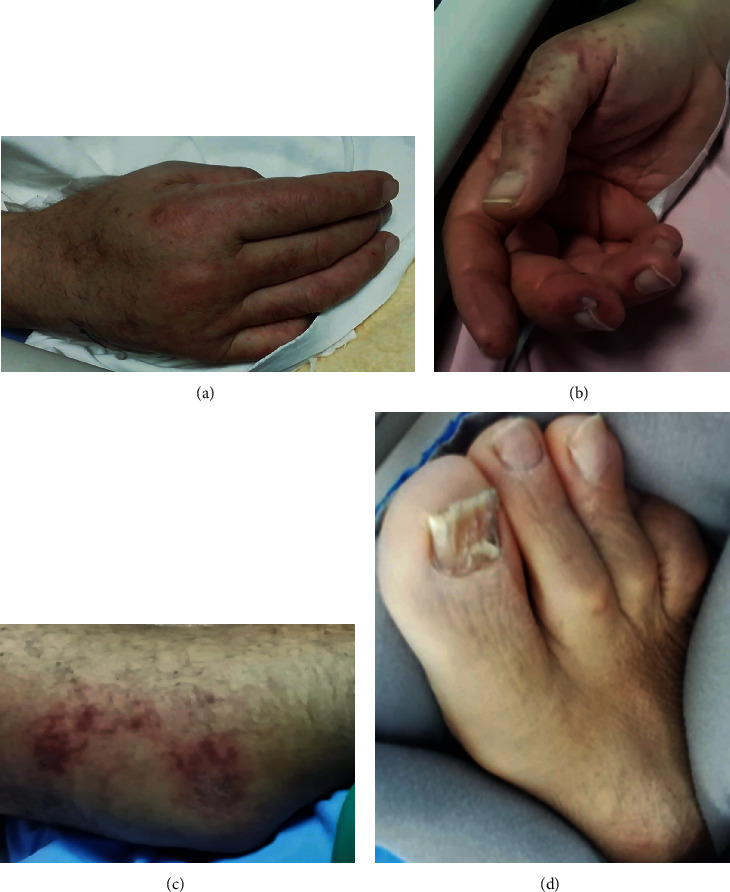
Improvement of lesions on dorsal (a) and palmar (b) area of hands, elbows (c), and lower limbs (d) seven days after initial presentation.

Due to the progression of the eruption, hydroxychloroquine treatment was withdrawn, and intravenous prednisolone was initiated at a dose of 0.5 mg/kg/day. The rash gradually improved (Figure 4) and was almost in complete remission after 10 days of treatment. At this time, the overall clinical picture also improved, and the patient was liberated from the ventilator. He gradually recovered and was discharged home on April 28, 2020, with no persistent or recurrent skin involvement.

## 3. Discussion

Respiratory viruses are well-known causes of exanthems. Coronaviruses have been known to be associated with petechial eruptions, albeit rarely [11]. Recent observations imply that SARS-CoV-2 is not an exception to the rule. Joob and Wiwanitkit have proposed that COVID-19 may manifest itself with a petechial skin rash [12]. Jimenez-Cauhe et al. described a patient that developed purpuric lesions, while being treated in the hospital for SARS-CoV-2 induced pneumonia [13].

The present case report describes a critically ill, SARS-CoV-2 positive patient with cutaneous, purpuric/livedoid lesions that were biopsied. The histologic examination revealed the presence of small-vessel, thrombotic (resembling disseminated intravascular coagulation, DIC) vasculopathy with very limited inflammation and absence of vasculitis. Similar findings have been described in coagulopathies, idiopathic thrombocytopenic purpura, and systemic DIC due to infections. However, clinical and laboratory evaluation ruled out the diagnoses of these disorders. Specifically, the patient scored less than 5 at the International Society for Thrombosis and Haemostasis diagnostic scoring system for DIC, thus, precluding the diagnosis of overt DIC [14]. Drug-related reactions were also included in the differential diagnosis. Although there are reports that link hydroxychloroquine to variable cutaneous adverse reactions such as erythema multiforme [15], morbilliform eruptions [16], and Steven's-Johnson syndrome [17], the majority of these have been described in patients with autoimmune underlying conditions, which were not present in our case. Additionally, hydroxychloroquine-related skin manifestations are usually noted after substantial cumulative doses (usually after a duration of treatment that exceeds 2-3 weeks) [18].

Viral rashes are frequent in clinical practice and usually present as a morbilliform exanthem, even though in the case of SARS-CoV-2 infection, a variety of skin manifestations has been described. In our case, the negative multiple PCR test on the skin samples does not exclude an indirect effect of viral activity at the cutaneous level. This hypothesis is also supported by our electron microscopy study of the skin samples, which did not reveal any viral inclusions in cutaneous cells or structures.

In a recent study that described the cutaneous symptoms of COVID-19 in 373 patients from Spain, it was reported that the purpuric/livedoid pattern was rare and encountered mostly in severely affected elderly patients [7]. Several other papers have also proposed that COVID-19 may manifest itself with a livedoid reticulated rash, often transient, and more pronounced in patients who are severely ill [12, 13]. Galván-Casas et al. also report that the rash usually appears after the development of pulmonary disease and has, therefore, limited diagnostic value. However, the authors suggest that it could be an indicator of vascular involvement in these patients [7]. The latter observation is in line with the histologic findings presented herein, as the findings from our histology samples were consistent with coagulative vasculopathy. It is, however, important to stress that the lesions described in our case cannot be classified as vasculitic, and it remains elusive whether described cases of vasculitis are distinct from the vasculopathic cases or present together as a continuum of vascular cutaneous injury.

In a case series described by Magro et al., pulmonary and cutaneous histologies from patients with severe COVID-19 were investigated, and it was reported that the purpuric skin lesions were associated with a pauci-inflammatory thrombogenic vasculopathy. In addition, the authors report deposition of C5b-9 and C4d, in both grossly involved and normal-appearing skin [9]. The histologic examination performed in our patient revealed similar findings; direct immunofluorescence demonstrated marked (+++) deposition of IgM, C3, and fibrinogen on vascular walls. IgA, C1q, and C4 deposition (++) were also present. These findings point strongly towards an intense complement activation that could potentially lead to a thrombotic-type coagulopathy. Complement activation may play a key role in the pathogenic process of COVID-19 and could be responsible for severe pulmonary morbidity; however, many questions remain unanswered. As the pathophysiological behavior of the host in response to the virus is currently being studied, it is becoming increasingly clear that the comprehensive and exhaustive examinations of all systems (including skin) are crucial elements of patient management.

The role of steroids and anticoagulants, whether protective—as this case suggests—or not, in this specific scenario of SARS-CoV-2 associated purpura should be explored in further studies.

In summary, we describe a case of transient livedoid/thrombotic lesions, most likely due to an occlusive, thrombotic microvasculopathy in a patient with severe COVID-19. To our knowledge, this is the first published case report, where electronic microscopy of pathologic skin was employed in a patient with SARS-CoV-2 infection diagnosis.

Due to the dramatic increase in incidence of COVID-19 and the alarming nature of vasculopathic lesions in critically ill patients, early recognition of such manifestations by dermatologists and intensive care physicians, along with a methodic work-up plan, is important to exclude more severe underlying coagulopathies. Further studies are required, to understand the vasculopathic effect of SARS-CoV-2 in the skin and other organs.

## Data Availability

The (figures of dermatologic lesions, histologic examination and electron microscopy study) data used to support the findings of this study are included within the article. For more information, contact with corresponding author, Anna Kyriakoudi (annkyr@gmail.com).
